# Neural mechanisms underlying the facilitation of naming in aphasia using a semantic task: an fMRI study

**DOI:** 10.1186/1471-2202-13-98

**Published:** 2012-08-10

**Authors:** Shiree Heath, Katie L McMahon, Lyndsey Nickels, Anthony Angwin, Anna D MacDonald, Sophia van Hees, Kori Johnson, Eril McKinnon, David A Copland

**Affiliations:** 1 University of Queensland, Language Neuroscience Laboratory, Centre for Clinical Research, Brisbane, Queensland, Australia; 2 University of Queensland, Centre for Advanced Imaging, St Lucia, Queensland, Australia; 3 ARC Centre of Excellence in Cognition and its Disorders, Department of Cognitive Science, Macquarie University, Sydney, New South Wales, Australia; 4 University of Queensland, School of Health and Rehabilitation Sciences, St Lucia, Queensland, Australia; 5 National Health and Medical Research Council Centre for Clinical Research Excellence in Aphasia Rehabilitation, Queensland, Australia

**Keywords:** Aphasia, Semantic verification, fMRI, Overt picture naming, Semantics

## Abstract

**Background:**

Previous attempts to investigate the effects of semantic tasks on picture naming in both healthy controls and people with aphasia have typically been confounded by inclusion of the phonological word form of the target item. As a result, it is difficult to isolate any facilitatory effects of a semantically-focused task to either lexical-semantic or phonological processing. This functional magnetic resonance imaging (fMRI) study examined the neurological mechanisms underlying short-term (within minutes) and long-term (within days) facilitation of naming from a semantic task that did not include the phonological word form, in both participants with aphasia and age-matched controls.

**Results:**

Behavioral results showed that a semantic task that did not include the phonological word form can successfully facilitate subsequent picture naming in both healthy controls and individuals with aphasia. The whole brain neuroimaging results for control participants identified a repetition enhancement effect in the short-term, with modulation of activity found in regions that have not traditionally been associated with semantic processing, such as the right lingual gyrus (extending to the precuneus) and the left inferior occipital gyrus (extending to the fusiform gyrus). In contrast, the participants with aphasia showed significant differences in activation over both the short- and the long-term for facilitated items, predominantly within either left hemisphere regions linked to semantic processing or their right hemisphere homologues.

**Conclusions:**

For control participants in this study, the short-lived facilitation effects of a prior semantic task that did not include the phonological word form were primarily driven by object priming and episodic memory mechanisms. However, facilitation effects appeared to engage a predominantly semantic network in participants with aphasia over both the short- and the long-term. The findings of the present study also suggest that right hemisphere involvement may be supportive rather than maladaptive, and that a large distributed perisylvian network in both cerebral hemispheres supports the facilitation of naming in individuals with aphasia.

## Background

An impairment of word finding or naming abilities (“anomia”) is the most common symptom of aphasia following brain injury and can be caused by a breakdown at any of the processing components involved in word production [1]. Importantly, naming performance can be improved in individuals with aphasia [2-4]. In fact, although naming treatments often utilize multiple exposures to repeated stimuli, a single application of a language task (“facilitation”) can have an effect upon subsequent naming performance [2-5]. This is also the case for unimpaired speakers, with previous behavioral research showing that the simple act of naming a picture once can speed subsequent naming of the same picture [6]. However, the mechanisms underpinning both the facilitation of unimpaired naming and treatment-induced improvement in individuals with aphasia remain uncertain. The present study, therefore, aimed to examine the effects associated with a specific semantic facilitation technique on subsequent picture naming performance in both individuals with aphasia and age-matched controls using fMRI.

In unimpaired speakers, improved naming performance following facilitation may reflect a form of repetition priming. These priming effects are thought to modify access to stored cognitive representations during first presentation, so that recognition and retrieval processes are enhanced on subsequent presentations [7,8]. It has been argued that the successful treatment of naming deficits in individuals with aphasia may rely on the same mechanisms that underlie priming in unimpaired naming [2,9]. Successful naming is a complex linguistic operation involving multiple sub-processes. Most theoretical models of spoken word production include at least two major levels of processing involved in picture naming: a lexical-semantic stage, where the meaning of a concept or picture is connected to its corresponding abstract lexical representation, and a phonological stage, where the relevant lexical-semantic unit is mapped onto the phonological word form [10-13]. Although underlying deficits and resulting symptom profiles differ markedly amongst individuals with aphasia, facilitatory techniques have traditionally targeted either the semantic or phonological level of processing [14,15]. This is due to the commonly held belief that semantic and phonological techniques target distinct components of the impaired word production system [16,17]. More recently, it has been proposed that these different types of tasks may also engage different neural regions [18-21]. It has been suggested that semantically based tasks are most effective for individuals whose primary area of deficit involves the semantic level of processing and, similarly, phonological tasks more effective for those with a phonological impairment [16,22]. However, this is not always the case and it is difficult to determine with any certainty the processing level at which facilitation is taking place [23]. Indeed both semantic and phonological processing are likely to occur during most language-related tasks, indicating that the differences between such techniques may be overstated [23,24].

It also appears that the beneficial effects resulting from phonologically- and semantically-focused tasks differ with respect to their longevity. Behavioral studies in both healthy controls and individuals with aphasia provide evidence for short-lived improvements (up to periods of several minutes) following a phonological task (but see [2]) and longer lasting benefits from a semantic task [4,8,25]. However, in the majority of studies utilizing a semantically focused task the phonological word form is provided in either the spoken or written modality, and it is thought that these longer-term effects may in fact be acting by strengthening the mapping operation between the processing levels [24]. This idea is supported by treatment and facilitation studies in aphasia that find limited benefits from semantic tasks that do not involve the relevant word form and which conclude that the phonological form is the critical element of such techniques [26,27]. While there is clearly a need to more comprehensively investigate the longevity of priming effects on naming, these findings support the proposal that tasks directly targeting either the lexical-semantic or phonological levels alone result in only short-term benefits, whereas longer lasting effects rely on activation of both semantics and phonology, by strengthening the mapping between them [24,25].

The use of functional neuroimaging in aphasia is beginning to provide more evidence regarding the neurocognitive substrates of treatment-induced improvement [28]. For example, studies utilizing a variety of linguistic tasks have shown that reorganization of function and recovery is dependent upon modulation of neural activity in spared left hemisphere language-related regions [29,30]. The role of the right hemisphere is less clear, with some research pointing towards a maladaptive functional reorganization to right hemisphere language homologues [31,32] and other studies proposing that both right and left hemisphere mechanisms contribute significantly to language recovery [33]. Various explanations have been put forward to account for inconsistencies across the literature, including the observation that the nature of relateralization may vary depending upon the size of the lesion [34,35]. Additionally, areas not traditionally associated with linguistic processing have been shown to support recovery from anomia in some individuals [14]. There is a growing body of literature attempting to detail the various neural mechanisms underlying improvement of language function, including research exploring the role of specific tasks used in treatment and the exact nature of their effects.

By way of example, several studies relevant to the current investigation have utilized fMRI to quantify neural changes prior to, and following, some form of intensive semantically focused treatment. A single case pilot study by Davis, Harrington and Baynes [36] used a decision-based semantic intervention and found improved covert verb generation associated with increased activation in several of their defined regions of interest following treatment, including the left inferior frontal gyrus and right posterior inferior temporal region. The authors suggest that increased activity in the left inferior frontal area may be due to their treatment task requiring selection of semantic information from competing alternatives. Increased activity in the right inferior temporal region, an area contralateral to the participant’s lesion, was taken to reflect a compensation of function by the right hemisphere homologue, or a change in strategy. Although one of the aims of their study was to determine whether word retrieval could be improved without explicit naming, silent production of responses during scanning can be problematic. It is difficult to ensure task compliance, and previous research has identified differences in brain activation patterns for covert and overt verbal responses [37] which may limit the findings. Additionally, the auditory cue used to prompt verb generation included the word form of the pictured item (e.g. for the picture ‘BALL’, the corresponding question was “What do you do with a ball?”). While the task was verb generation, participants heard the word form of the pictured item and may have attempted to covertly name the picture, making it difficult to isolate any effects to semantic processing.

Fridriksson et al. [14] also utilized a semantic treatment. Their study examined the neural correlates of improved naming by comparing both a semantic and phonological intensive treatment in three individuals with aphasia, two of whom suffered from non-fluent type aphasia. Their semantic treatment took the form of a detailed auditory cueing hierarchy that did not include the phonological form until the final cue, which for both treatment hierarchies involved auditory repetition of the target item. All three participants benefitted behaviorally to varying degrees from both techniques and generally showed increased activity within neural regions not traditionally linked to language processing. The authors proposed that their findings of activation in regions not normally associated with language processing (e.g., right entorhinal cortex and bilateral precuneus) may represent a form of compensatory cortical adaptation, as opposed to any specific repair of the normal language network [14,38].

Researchers examining brain activity before and after treatment of aphasia commonly face the difficulties inherent in interpretation of imaging data when comparing patterns of activity during poor performance prior to treatment, to good performance following treatment. A complementary way to advance understanding of the mechanisms underlying treatment is to look more directly at the brain activity occurring during performance of a particular treatment task. Perani et al. [39] compared activation during both a phonological and semantic covert verbal fluency task in five participants with aphasia. For patients with good recovery, the principal finding regarding the semantic task was activation in the left inferior frontal gyrus, possibly reflecting the use of an effective and effortful lexical retrieval strategy. However, in the case of those patients with impaired performance, there was extensive bilateral prefrontal activation, which may have indicated increased “mental effort” required for retrieval attempt rather than retrieval success [39].

A more recent study compared a semantic judgment task to a control task [34] in eight participants with aphasia who differed in terms of site of lesion. The results of the experiment indicated that all individuals without damage to the left inferior frontal region activated the left inferior frontal gyrus, similar to their control group of eight individuals. They concluded that successful completion of a complex semantic judgment task required the inhibition of competing items. In contrast, those participants who did have some inferior frontal lesion involvement activated contralateral regions, in addition to perilesional left frontal regions. It should be noted that the semantic judgment task used in their study involved the presentation of written words and therefore included the orthographic form of stimuli. The presence of the word form means that orthographic, phonological and lexical-semantic processes were likely to be engaged during performance of the task.

Although no definitive picture has emerged regarding the neural correlates associated with performance of particular language tasks used in treatment, or the mechanisms involved in the recovery of language following treatment, together these studies demonstrate that semantic interventions can result in positive effects for some individuals. Facilitation-induced improvement in performance, however, has not been as extensively investigated. Studies exploring the facilitatory effects of specific treatment tasks upon subsequent naming are principally limited to behavioral studies [2-5]. The examination of naming facilitation in conjunction with neuroimaging may increase our knowledge regarding the relationship between facilitation and treatment and may enable us to determine how specific treatments are having their effects at a neurocognitive level. Moreover, the ability to measure the bases of successful naming facilitation may provide a method for predicting positive treatment outcomes in individuals with anomia [3].

Therefore, in the present study we aimed to determine the behavioral and neurocognitive effects of picture naming facilitation by a prior semantic task in both unimpaired speakers and individuals with aphasia using fMRI. Unlike other studies, we did not utilize fMRI before and after a semantic task, or simply have participants perform the task within a scanner. Instead, participants performed a semantic facilitation task and then fMRI was used to investigate the differences in neural activation between facilitated and unfacilitated conditions during subsequent picture naming. To minimize the involvement of any phonological processing, the semantic questions used during the facilitation task did not include the phonological word form and participants were not required to produce the word form in response. We also sought to determine the longevity of any effects by manipulating the timing of the prior semantic task. Consequently, the long-term condition was facilitated several days prior to subsequent naming, and the delay between facilitation and subsequent naming for the short-term condition was a period of several minutes. Importantly, the three main picture naming conditions of interest (long-term facilitated, short-term facilitated and unfacilitated) were presented within a single scanning session. To our knowledge, no other neuroimaging study has utilized this specific design to test the underlying cognitive mechanisms involved in successful picture naming following facilitation with a targeted semantic task in both unimpaired speakers and individuals with aphasia.

It was hypothesized that participants with aphasia would engage similar regions to age-matched controls or their right hemisphere homologues during successful naming of previously facilitated items, with associated modulation of activity primarily in regions linked to semantic processing. This would be evident by minimal involvement of areas linked to phonological processing and significant changes in activation within a combination of spared left hemisphere semantic regions (or areas close to damaged semantic regions), and/or in the right hemisphere homologues of those regions. Further, we tested the hypothesis that our targeted facilitation task would result in relatively short-term effects upon subsequent picture naming, based on previous behavioural observations [8,25,40]. Findings in line with this hypothesis would indicate that temporary facilitation was occurring selectively at the lexical-semantic level, as opposed to strengthening the links between the lexical-semantic and phonological levels of processing. It was also hypothesized that any longer lasting effects would involve areas linked to both lexical-semantic and phonological processing, indicating that more durable facilitation from a semantic task is dependent upon a strengthening of connections between the two levels of processing [24].

## Methods

### Participants

All participants were right handed and reported English as their first language. Each participant was tested for visual acuity, screened for cognitive impairment by administration of the Mini-Mental State Examination [41] and for depression using the Geriatric Depression Scale [42]. For both controls and participants with aphasia, exclusionary criteria included significant hearing loss (as identified by pure tone audiometry screening), a history of alcohol abuse, mental illness, or any other neurological disease or disorder. Exclusionary criteria also included the presence of any contraindications for magnetic resonance imaging. Other than reimbursement of travel costs, participants received no direct financial benefit from involvement in the study. Ethical approval was obtained from the University of Queensland and the Research Ethics Committees of five major metropolitan hospitals in Brisbane, Australia (including the Royal Brisbane and Women’s Hospital, the Princess Alexandra Hospital, the Queen Elizabeth II Jubilee Hospital, the Logan Hospital and the Prince Charles Hospital). Additionally, all participants gave informed written consent under an approved University of Queensland Medical Research Ethical Review Committee protocol and in accordance with the Declaration of Helsinki, and express consent to publish any relevant clinical information and data.

#### Control participants

Full details of the experiment conducted with control participants has previously been reported [43]. Nineteen healthy older adults participated in the study, with data from one participant removed due to a high percentage of slow reaction times (36.4% of correct responses > 1500 ms). For the remaining 18 (10 female) controls, the average age was 56.2 years (SD = 10.4, range 38 to 74 years) and average tertiary educational level was 16.6 years (SD = 3.0, range 12 to 22 years). The average age of controls did not differ significantly from that of the participants with aphasia (t(25) = 0.312, p = 0.684).

#### Participants with aphasia

**Figure 1:**
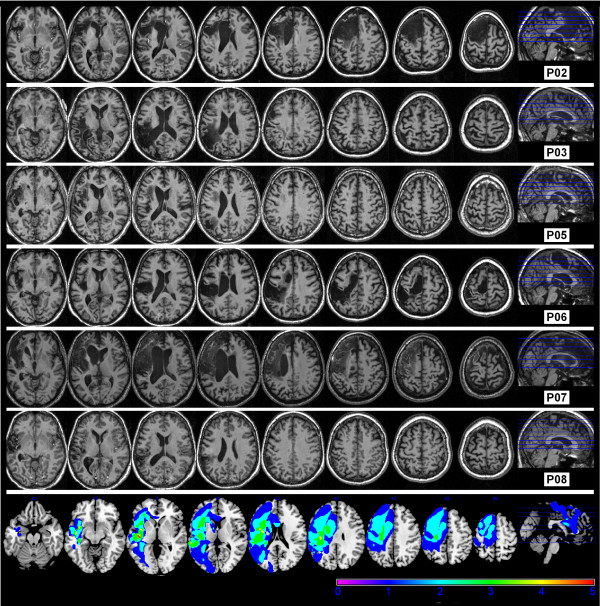
**Lesion characteristics.** Axial slices from a 3D T_1_ weighted MRI at 10 mm intervals for each participant with aphasia (starting at z = −6) and a lesion overlay map.

The presence and classification of aphasia in participants was determined based on the results of both the Comprehensive Aphasia Test [44] and the Western Aphasia Battery [45]. Exclusionary criteria included the presence of any perceptual deficit, severe apraxia or severe dysarthria. Eight individuals with chronic aphasia following a single left-hemisphere cerebrovascular accident participated. The data from two male participants (P01 and P04), however, was removed prior to analysis due to the fact that one participant did not classify as aphasic based upon assessment results and generalized sulcal atrophy was identified for the other participant on structural magnetic resonance imaging (MRI). For the remaining six participants with aphasia (five female), the average age was 57.6 years (SD = 11.2, range 39 to 71 years) and average formal education level was 12.5 years (SD = 2.3, range 10 to 16 years). Table 1 sets out relevant demographic and clinical details. Additionally, for each participant eight axial slices from a T_1_ weighted MRI are shown in Figure 1, with a corresponding lesion overlay map. An automated method was used to identify lesions [46]. Assessment battery results and possible levels of impairment for each participant are set out in Table 2. Four of the six participants were classified with an anomic aphasia, one with a Wernicke’s type aphasia (P03) and one with conduction aphasia (P08) according to the Western Aphasia Battery [45]. 

**Table 1:** Demographics and clinical details of participants with aphasia

	**P02**	**P03**	**P05**	**P06**	**P07**	**P08**
Gender	F	F	M	F	F	F
Age	39	71	59	66	52	59
Education	14	10	13	12	16	10
Time Post-Stroke	12 years; 10 months	14 years; 1 month	3 years; 9 months	6 years; 3 months	3 years; 11 months	2 years; 4 months
Lesion Volume (cm^3^)	135.68	83.13	39.51	122.09	166.30	22.68
Lesion Involvement	IFG (opercularis)	Supramarginal Gyrus	IFG (opercularis)	Middle Frontal	Inferior Frontal	Superior Temporal
	IFG (triangularis)	Angular Gyrus	Insula	Supramarginal Gyrus	Middle Frontal	Middle Temporal
	Middle Frontal	Superior Temporal	Caudate Nucleus	Superior Temporal	Superior Frontal	Supramarginal Gyrus
	Superior Frontal	Middle Temporal	Putamen	Heschl’s Gyrus	Rolandic Operculum	Hippocampus
	Rolandic Operculum	Heschl’s Gyrus	Hippocampus	Insula	Insula	
	Insula	Rolandic Operculum		Rolandic Operculum	Superior Temporal	
	Cingulate (middle)	Insula		Supplementary Motor	Heschl’s Gyrus	
	Cingulate (anterior)	Superior Occipital		Paracentral Lobule	Supramarginal Gyrus	
	Caudate Nucleus	Middle Occipital		Cingulate (middle)	Putamen	
	Precentral				Precentral	
	Supplementary Motor				Postcentral	

**Table 2:** Behavioral results for participants with aphasia

	**Max**	**P02**	**P03**	**P05**	**P06**	**P07**	**P08**
**Pre-tests:**							
- total accuracy		74.8%	49.5%	81.2%	81.4%	88.8%	53.0%
- phonological errors		1.6%	13.1%	11.9%	9.0%	3.9%	25.9%
- semantic errors		25.8%	15.4%	47.1%	42.3%	41.4%	9.6%
**WAB**:							
- spontaneous speech	20	18	14	17.5	18	18	17
- comprehension	200	200	111	181	199	198	166
- repetition	100	86	68	70	97	98	46
- naming/word finding	100	91	71	87	92	96	85
- aphasia quotient	100	91.4*	66.8*	84.5*	93.7*	94.6	76.8*
- classification		Anomic	Wernicke’s	Anomic	Anomic	Anomic	Conduction
**BNT:**	60	42*	24*	55	41*	55	33*
**P&PT**:							
- three pictures	52	50	45*	52	50	50	51
- spoken word, two pictures	52	52	45*	50	52	50	48*
**CAT**:							
- spoken comprehension	66	62	48*	56*	60	61	48*
- repetition (5 subtests)	74	60*	43*	48*	70	60*	26*
- real words	32	30	22	26	32	29	17
- complex words	6	4	3	4	6	6	1
- non-words	10	6	6	6	8	5	0
- naming	58	47*		52*	53*	54*	46*
- fluency	un	15		24	15	18	20
- reading	70	64		64	68	58*	57*
- spoken picture description	un	13*		36	35	43	40
**Possible Levels of Impairment**		▪ semantics to phonology and/or phonological form	▪ semantics▪ semantics to phonology and/or phonological form	▪ semantics to phonology and/or phonological form	▪ semantics to phonology and/or phonological form	▪ semantics to phonology and/or phonological form	▪ semantics to phonology and/or phonological form
							▪ post-phonology
**Facilitation Task Accuracy:**							
- total accuracy to targets		90.6%	50.6%	88.8%	96.0%	95.9%	79.9%
- to long-term targets		94%	45.9%	95.3%	100%	97.9%	81.9%
- to short-term targets		84%	60%	76%	88%	92%	76%
- to ‘no’ response fillers		100%	66.6%	66.6%	79.1%	100%	91.6%

Max = maximum raw score possible on each subtest; un = unlimited possible maximum score. * indicates score falls below normal range with reference to available normative data for the Western Aphasia Battery (“WAB”) [45], the Pyramids and Palm Trees Test (“P&PT”) [84], the Comprehensive Aphasia Test (“CAT”) [44], and in the case of the Boston Naming Test (“BNT”) with reference to Australian normative data [85].

### Stimuli

Stimuli were sourced from a digital photographic database (Hemera Photo-Objects, Hemera, Hull, Canada), as well as other royalty free digital stock photographs, and included items from ten broad semantic categories (people, animals, objects, food, clothing, vehicles, places, tools, body/animal parts and natural phenomena). Fifteen non-objects were also produced (CorelDRAW Graphics Suite II, Corel Corporation, Ottawa, Canada) for display at the beginning of each experimental block. Each non-object was pixelated to the point of being unrecognizable, but maintained the same luminance and properties as experimental stimuli. All images were grey-scaled photographs of approximately the same size (no larger than 500 x 420 pixels) with a white background (600 x 600 pixels). The images had an average luminance of 223.68 candela per m^2^ (SD = 19.84, range 151.44 and 253.67). Mean reaction times and percentage name agreement data for each target word was sourced from the International Picture Naming Project (IPNP) database [47]. Age of acquisition norms were obtained from Morrison, Chappell, and Ellis [48] and frequency counts from the CELEX lexical database [49]. Imageability ratings were obtained from the Medical Research Council psycholinguistic database [50].

A large group of 476 pictures was compiled, from which a subset of 75 experimental stimuli were chosen (60 for controls) following two pre-test sessions (a single pre-test session for controls). A larger subset of items was chosen for the individuals with aphasia, who were more likely to have difficulties naming, so that a similar number of successful trials were produced by both participant groups. These were then divided into three sets with reference to their category of facilitation (short-term facilitated, long-term facilitated, or unfacilitated). Each set contained 25 items (20 items for controls). Assignment of sets to conditions was counterbalanced across control participants. An additional set of 20 non-critical filler items was also included for control participants, so that the number of trials within the scanning session for both subject groups was identical. All sets were matched on the basis of IPNP reaction time [47], frequency [49], number of phonemes and number of syllables. For controls, sets were also matched for age of acquisition [48] and imageability [50]. For participants with aphasia facilitated sets were also matched for accuracy and, in the case of those with milder naming deficits where items incorrectly named in only one pre-test were required to be included, with regard to the participant’s own average reaction time across both pre-tests. Additionally, the Edinburgh Associative Thesaurus [51] was utilized to ensure no stimulus item within a set was the most commonly produced associate of any other item within that set.

For participants with aphasia, it was important to ensure that the items chosen to be the target of facilitation were difficult to name. For this reason, the short-term facilitated and long-term facilitated sets of stimuli were sourced from those items that participants consistently found difficult to name during both pre-tests (inaccurate, or no response within 3 seconds). Items with long reaction times across both pre-tests and items incorrectly named in only one pre-test were also included in facilitated sets for those participants with milder naming deficits. The items included in the unfacilitated set, however, were chosen from those items that were able to be named consistently across both pre-test sessions. This unfacilitated baseline condition could be compared to those items that were consistently difficult to name and that were to be the target of facilitation, thereby enabling examination of whether facilitation recruits neural regions distinct from those already engaged during successful naming. To clearly differentiate the baseline condition for each group of participants, we refer to the unfacilitated set for controls as “unfacilitated” and to the unfacilitated set for participants with aphasia as “known”.

### Procedure

The study employed a quantitative case series design, utilizing a repeated measures analysis with a single independent factor of facilitation (short-term facilitated, long-term facilitated, or unfacilitated/known), and with behavioral results and neural activity as measured by fMRI as primary dependent variables of interest. Participation involved pre-test sessions (one session for controls and two for participants with aphasia), two facilitation sessions and an experimental fMRI scanning session. For the participants with aphasia, a subsequent follow-up session was also conducted. During the pre-test sessions, each item in the large collection of 476 pictures was randomly presented for naming. Two pre-test sessions were conducted for participants with aphasia, due to the inconsistency often associated with anomic symptoms. The results from both sessions provided a more stable baseline and allowed identification of items that were consistently difficult to name and consistently able to be named.

**Figure 2:**
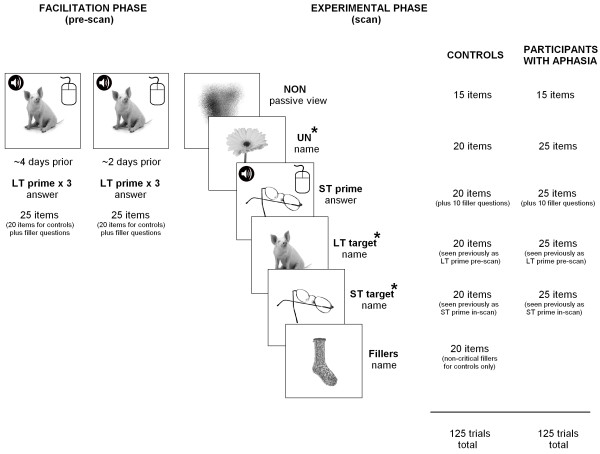
**A summary of the presentation of randomized stimuli.** Facilitation phase: one set of pictures were presented three times on two separate occasions (six times total), simultaneously with a semantic auditory question, for yes/no response by computer mouse button (LT prime). Experimental phase (during scan): the long-term facilitated set were presented again for naming (LT target); one set of pictures were presented twice - once as a prime along with an auditory question for yes/no response (ST prime) and then presented again (6 to 8 trials later) for naming (ST target); and one set of unfacilitated pictures were also presented once for naming (UN) (referred to as the “known” condition for participants with aphasia). For control participants, an additional set of unfacilitated non-critical fillers were also presented.

The experiment was conducted in two phases over the course of approximately three weeks (refer to Figure 2). In the facilitation phase all participants were required to complete two facilitation sessions, being no more than three days apart. The behavioral task used for the facilitation sessions was created using E-Prime (version 1.1) (Psychology Software Tools, Pittsburgh, PA). During each of these two sessions the stimuli representing the long-term facilitated condition were presented three times, each time in a different random order (i.e., six times in total). A single trial consisted of a fixation point (+) displayed for 1,500 ms, followed by a picture for a period of 3,000 ms. Each picture was presented simultaneously with an auditory question relating to the semantic properties of the item. The auditory semantic questions for each image were spoken by a female voice and digitally recorded in a soundproof recording studio at 44100 Hz, mono, 32 bit. The semantic questions for critical items required a positive response to semantic questions, to ensure the semantic properties associated with each target item were engaged. Additional questions requiring a no response were randomly interspersed within the facilitation session, to ensure unpredictability of response type. Participants were required to respond by computer mouse button. Both the hand used to respond, and the mouse button representing yes and no, was counterbalanced across participants and then held constant across sessions. No feedback was given regarding accuracy of responses. Additionally, the questions did not contain any reference to the word form of the relevant item. For example, for the picture ‘CAT’, the question would be “Does it purr?”, as opposed to “Does a cat purr?”. In other words, participants did not hear, and were not required to produce, the phonological word form during facilitation. Also, no other target item was included in the question (e.g., the question for the picture of a sock did not include the words “shoe” or “foot” if these were also target items). Semantic questions were based upon Garrard, Lambon Ralph, Hodges, and Patterson’s [52] classification types. They took the form of either sensory (e.g., “Is it wet?”), functional (e.g., “Is it worn?”) or encyclopaedic (e.g., “Is it found in the sky?”) questions. Types of semantic questions were evenly distributed across short-term and long-term conditions. Following completion of both facilitation sessions, participants had been randomly presented with each picture item from the long-term facilitated set, together with its corresponding auditory semantic question. A single question was asked for each critical target item, a total of six times. Although a single prior exposure can affect subsequent naming, multiple exposures to long-term facilitated items is in line with current treatment practices [53]. 

The experimental phase of the study involved an fMRI scanning session, where all three sets of stimuli were presented for naming (see Figure 2). For all participants the fMRI session was conducted in 3 runs, with 2 runs of 40 individual trials and one run of 35 trials, resulting in 125 trials in total. A single trial lasted 14.7 s and consisted of a 250 ms period of blank screen, followed by a target picture displayed for a period of 3 seconds. This was followed by a blank screen for 9.45 s. The commencement of the following trial was indicated by a fixation point (+) displayed for 2 seconds. The task for the scanning session was created using Microsoft Visual Basic 6.0 (Microsoft Corporation, Redmond, WA). Stimuli were back-projected onto a luminous white screen and viewed through a mirror mounted on the head coil which was subtended approximately 10 degrees of visual arc. Responses were digitally recorded (sampling rate 11 kHz) with an optical single channel noise cancelling microphone (FOMRI, Optoacoustics Ltd., Or-Yehuda, Israel) and auditory stimuli were played through a pair of Piezoelectric MR compatible headphones (MR Confon, Madeburg, Germany).

The items forming the long-term condition, which had previously been presented during the facilitation sessions, were presented again in the scanner to investigate any longer lasting facilitation effects. The short-term facilitated set of items was presented twice within the scanner, in different random order and separated by a period of no more than 3 minutes. They were presented firstly as a prime, along with an auditory semantic question for a yes/no button response, and then presented again (within a lag of 6 to 12 trials, average 10) as a target for naming, to enable investigation of any short-term facilitation effects. Similar to the facilitation phase, ten semantic questions requiring a negative response were interspersed with critical short-term prime items and subsequently presented again for naming. The set of unfacilitated/known items was also presented once within the scanner as a baseline for comparison purposes. Stimuli were presented pseudo-randomly in blocks of five trials per condition (long-term facilitated condition, short-term facilitated prime and target conditions, and unfacilitated/known condition) throughout the scanning session, to ensure participants were aware of what task was required for each item and to minimize any effects of constant task switching. At the commencement of each block, task instructions were displayed on the screen as either the word “Name” (for critical and filler target items) or the word “Answer” (for short-term facilitated prime questions and filler questions requiring a no response).

Finally, for the participants with aphasia, a subsequent follow-up session occurred approximately one week after the scanning session. This session involved the random presentation of all experimental stimuli once for naming, to provide a measure of post-facilitation improvement. Unfortunately, two participants with aphasia (P05 and P06) were unavailable for follow-up sessions.

### Image acquisition

A 4-Tesla Bruker MedSpec MRI (Bruker Medical, Ettlingen, Germany) was used to acquire images, with a transverse electromagnetic head coil [54] utilized to enhance imaging resolution at high field strength. Gradient-echo, echo planar images (GE-EPI) (matrix size of 64 x 64; repetition time (TR) 2100 ms; echo time (TE) 30 ms; 90° flip angle; field of view 230 mm) with an interleaved gradient acquisition sequence were acquired in 36 axial planes with in-plane resolution of 3.6 mm and slice thickness of 3 mm (0.6 mm gap). A behavioral interleaved gradient design was employed, primarily to avoid artefacts associated with head movement during overt naming. The design also allows participants to hear the auditory stimuli with minimal scanner noise during picture presentation, and permits the recording of verbal responses and accurate reaction times [55,56]. Only slice gradients were applied during the critical interval, with radiofrequency intact to maintain steady state magnetization [56]. For the 10.5 s in which the blank screen (8.5 s) and fixation point (2 s) were displayed, image acquisition occurred to capture the blood oxygen level-dependent (BOLD) response for that naming trial. A total of 625 GE-EPI volumes were acquired over three runs. To allow magnetization to reach steady state, the first five volumes (the first 10.5 s) in each run prior to the presentation of stimuli were discarded. Prior to GE-EPI acquisition, a PSF (point-spread function) mapping sequence was acquired, allowing the distortion in geometry and intensity to be corrected in the time series data [57]. Within the same session a three-dimensional T_1_ weighted MP-RAGE (magnetization-prepared rapid gradient-echo) was acquired (matrix size of 256 x 256; TR 2200 msec; TE 2.99 msec; inversion time (TI) 900 msec; 9° flip angle; resolution 1.0 x 1.0 x 1.0 mm³; field of view (FOV) 256 mm), as well as a T_2_ weighted FLAIR (fluid-attenuated inversion recovery) image (matrix size of 256 x 256; TR 8000 msec; TE 98 msec; TI 2200 msec; 180° flip angle; resolution 0.9 x 0.9 mm in-plane; 19 x 6 mm slices; FOV 230 mm) for monitoring of participant stability, identification of any white matter hyperintensities and to confirm lesion volume.

### Data processing

Behavioral data for short-term facilitation prime trials in the experimental phase and, in the case of control participants, data associated with non-critical filler items were excluded. Critical naming trials to which no response, or an incorrect response, was recorded from participants were also excluded (35.7% of responses for participants with aphasia and 5.04% for controls). Statistical Parametric Mapping (Version 5) software (SPM5, Wellcome Department of Cognitive Neurology, London, UK) was used with MATLAB 2009a (The MathWorks Inc., Natick, MA) to process and analyse the imaging data. During spatial preprocessing the image time series were first realigned using rigid body motion correction with INRIAlign [58]. The mean EPI image generated from the realigned series for each participant was coregistered with the T_1_ image acquired in the same session. The T_1_ image was then normalized to the standard Montreal Neurological Institute (MNI) [59] atlas T_1_ weighted template and these transformations were applied to the realigned EPI time series. Normalised volumes (3.0 x 3.0 x 3.0 mm^3^) were spatially smoothed using an 8 mm full-width half-maximum Gaussian kernel. Due to utilization of a behavioral interleaved gradient design, the partial time course of fMRI data was modeled in the general linear model (GLM) using a finite impulse response basis function. The onsets and durations were chosen to reflect the expected peak of the BOLD response and age was included as a covariate in the GLM. All short-term primes and incorrect responses were also treated as trials of no interest in the GLM analysis.

### Data analysis

For participants with aphasia, single subject whole brain analyses were conducted with a height threshold of p < 0.01 and a minimum cluster size of greater than 20 voxels. An automated approach was used to delineate brain lesions [46] and lesion volumes were calculated using fslstats (FMRIB Software Library - http://www.fmrib.ox.ac.uk/fsl).

As reported in [43], group level whole brain analyses (*p* < 0.001) were then conducted for controls. A correction for multiple comparisons was calculated using 3dClustSim, implemented in AFNI (Analysis of Functional Neuroimages, National Institute of Mental Health, Bethesda, MD) [60]. Adopting a height threshold of *p* < 0.001, a FWE (family wise error) rate of *p* < 0.05 was achieved with a minimum cluster threshold of 23 contiguous voxels. For all imaging analyses, automated anatomical labeling software [61] was used to identify the neuroanatomical location of peak maxima for specific contrasts.

## Results

### Control participants

Results for control participants have been reported separately elsewhere (refer to [43]). The reaction time data analyses were conducted on correct responses, with pairwise comparisons identifying significant differences between all conditions (*p* < 0.05). Both short- and long-term facilitated items were named significantly faster than unfacilitated items and short-term items significantly faster than long-term items. Due to the mean percentage accuracy being above 97% for all conditions, no main effect of condition was found upon analysis of the accuracy data. There was a trend for long-term facilitated items to be named most accurately, with short-term items named more accurately than unfacilitated items.

Group level whole brain analyses were then conducted on fMRI data from the control group (refer to Table 3). An increase in activity for short-term facilitated items was identified within certain language-related regions: in the right lingual gyrus (extending to the precuneus region) when compared to unfacilitated items and in the left inferior occipital gyrus (extending to the left fusiform gyrus) when compared to long-term facilitated items. For main contrasts of interest, changes in activity were also identified in the primary motor and somatosensory cortices. In the left precentral gyrus greater activity was identified for long-term facilitated items than for short-term facilitated items. A decrease for short-term facilitated items was found in the right postcentral gyrus and for long-term facilitated items in the right precentral gyrus when compared to unfacilitated items.

**Table 3:** MNI coordinates of peak activation for control participants

**Contrast description and anatomical label**	**Volume**	**x**	**y**	**z**	**Z-score**
Short-Term > Unfacilitated					
- right lingual gyrus extending into precuneus	29	9	−42	6	4.20
Short-Term > Long-Term					
- left inferior occipital gyrus extending into left fusiform gyrus	36	−39	−69	−9	4.19
Long-Term > Short-Term					
- left precentral gyrus	28	−30	−9	42	3.98
Unfacilitated > Short-Term					
- right postcentral gyrus	24	30	−42	69	4.15
Unfacilitated > Long-Term					
- right precentral gyrus	28	57	−12	45	3.45

Peak activation from the whole brain analyses (p < 0.001) for clusters with a minimum of 23 contiguous voxels.

### Participants with aphasia

**Figure 3:**
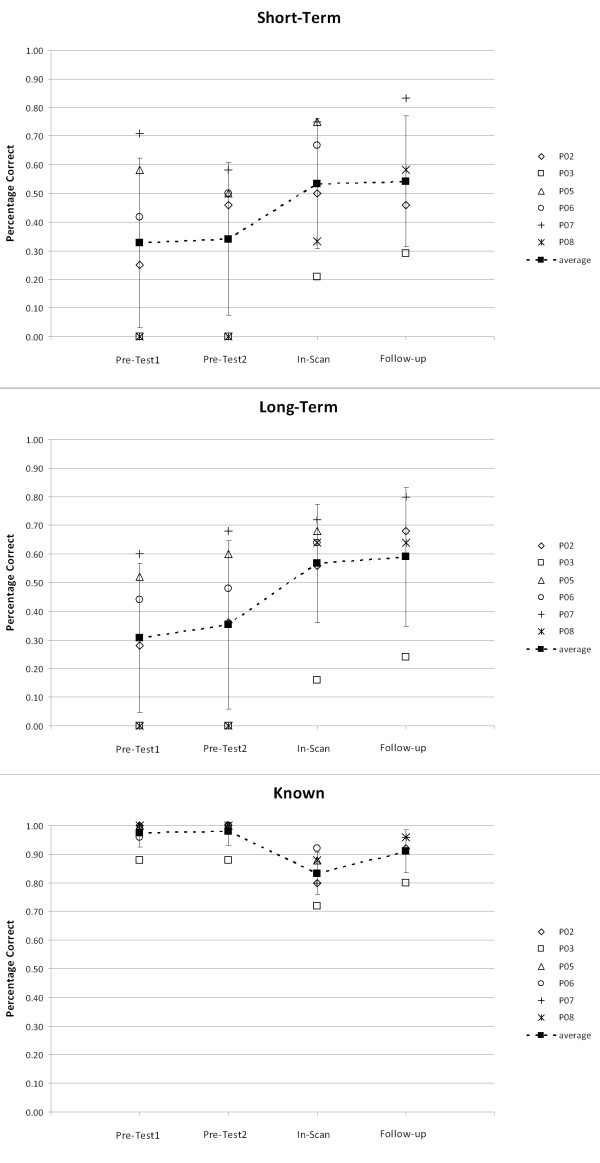
**Facilitation effects in accuracy data for participants with aphasia.** Facilitation effects in percentage accuracy data for each participant with aphasia for all conditions. Refer to supplementary material Figure S1 for individual graphs.

As an indication of participants’ ability to perform the facilitation task, accuracy for semantic questions during facilitation sessions (for long-term items) and at prime presentations during scanning (for short-term prime items) are reported in Table 2. Percentage accuracy data for naming for all conditions from the pre-test sessions and experimental phase of the study are shown in Figure 3. A weighted Wilcoxon One-Sample test was used to identify whether accuracy performance at pre-facilitation (Pre-Tests 1 and 2) differed from accuracy post-facilitation (In-Scan and, where available, Follow-Up) for each condition. Refer to supplementary material Figure S1 for individual graphs for each participant. P05 did not show a significant change in accuracy performance for facilitated conditions. For the remaining participants: P02 showed a significant positive change for long-term facilitated items, P06 and P07 for short-term facilitated items, and P03 and P08 for both long-term and short-term facilitated items (p < 0.05, one-tailed). As P05 and P06 were unavailable for follow-up data collection, we compared level of performance at both pre-test sessions to the single, in-scanner post-test for these two participants. A Mann–Whitney U test was subsequently conducted to determine whether the magnitude of change from pre- to post-facilitation differed across conditions for each participant. Results showed no overall significant difference in change of accuracy performance from pre- to post-facilitation between the short-term facilitated and long-term facilitated condition for any participant. Finally, a significant decrease in performance for participants was identified pre- to post-facilitation for known items, with the exception of P05 and P06.

**Figure 4:**
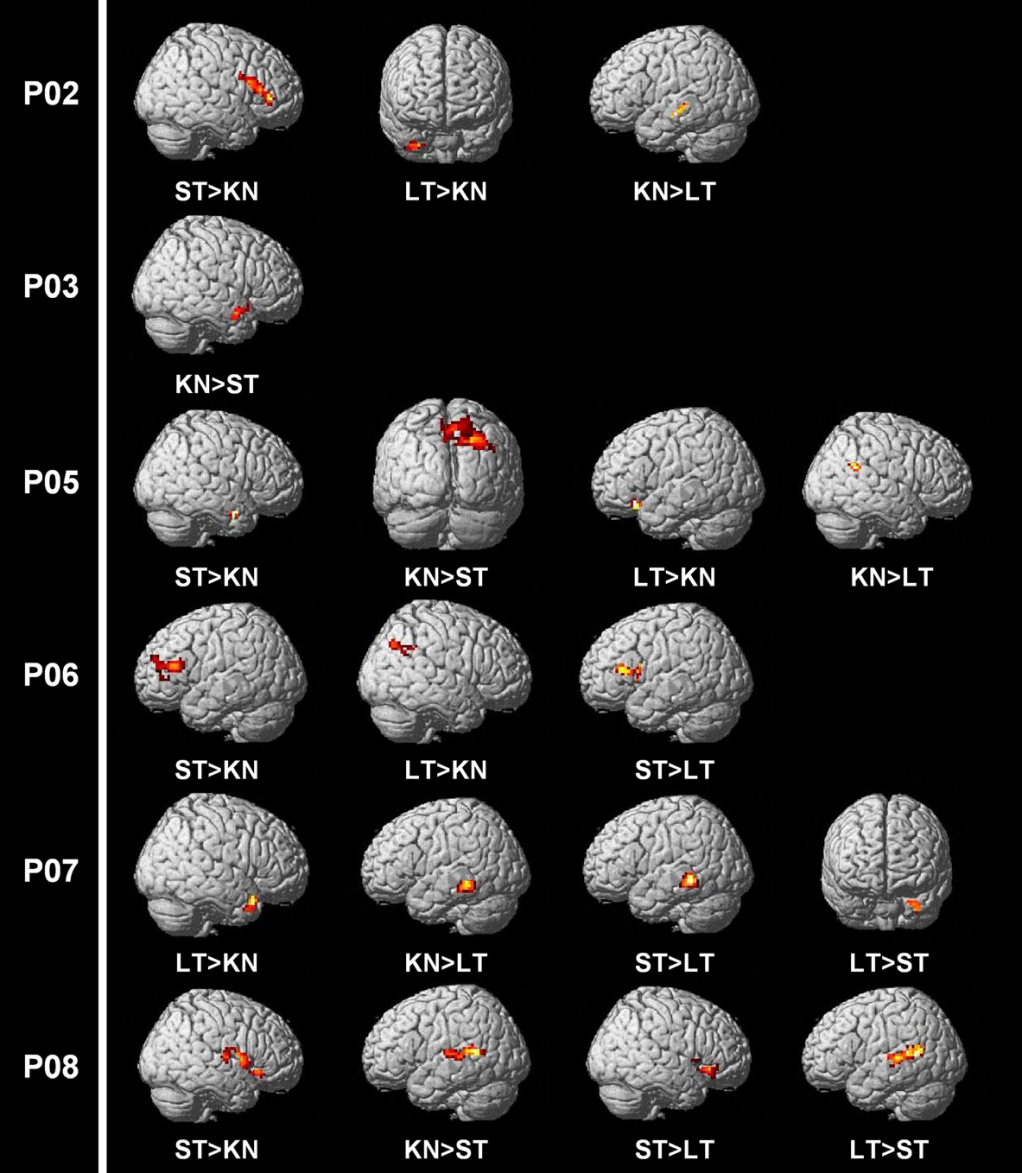
**Whole brain analyses for participants with aphasia.** Most significant results in language-related regions for relevant contrasts of interest (p < 0.01, greater than 20 contiguous voxels) based on highest Z-score and/or largest cluster size (refer to highlighted contrasts in Table 4).

Full whole brain analyses results are set out in supplementary material Table S1 and those identified in language-related regions are reported below (refer to Table 4). The most significant of these language-related results (based on largest cluster and/or highest z-score) are displayed in Figure 4. A case-series of summarised results is reported, as group analyses of people with aphasia can be problematic [31,62]. As incorrect and no response trials were excluded, these results represent modulation of activity during accurate picture naming for each condition. 

**Table 4:** Summarized whole brain results (p < 0.01, greater than 20 contiguous voxels) setting out significant language-related results for each participant with aphasia

**Region**	**P02**	**P03**	**P05**	**P06**	**P07**	**P08**
**Left Phonological**						
Inferior Frontal Gyrus (opercularis)						
Superior Temporal Gyrus						**LT > ST**
Supramarginal Gyrus						
**Right Homologue of Phonological Region**						
Inferior Frontal Gyrus (opercularis)						**ST > KN**
Superior Temporal Gyrus						ST > LT
Supramarginal Gyrus	LT > KN			ST > KN		ST > KN
**Left Semantic**						
Inferior Frontal Gyrus (orbitalis)	ST > KN		**LT > KN**			ST > LT
Inferior Frontal Gyrus (triangularis)				**ST > KN**		
				LT > KN		
				**ST > LT**		
Superior Temporal Pole						
Middle Temporal Gyrus	ST > KN (pole)			ST > KN	**KN > LT**	**KN > ST**
	**KN > LT**			LT > KN	**ST > LT**	
Inferior Temporal Gyrus					LT > ST	
Angular Gyrus						
**Right Homologue of Semantic Region**						
Inferior Frontal Gyrus (orbitalis)	LT > KN					
Inferior Frontal Gyrus (triangularis)	**ST > KN**			ST > KN	KN > LT	**ST > LT**
					ST > LT	
Superior Temporal Pole		**KN > ST**			LT > KN	
					LT > ST	
Middle Temporal Gyrus	ST > KN (pole)		**ST > KN**		**LT > KN**	ST > LT
					KN > LT	
					ST > LT	
Inferior Temporal Gyrus	**LT > KN**		KN > LT	LT > KN	**LT > ST**	
Angular Gyrus			**KN > ST**	**LT > KN**		ST > LT
			**KN > LT**			

Refer to full tabulated results (supplementary material Table S1) for cluster volume, MNI coordinates and Z-scores. Contrasts in bold represent the most significant results for each participant based on highest Z-score and/or largest cluster size.

KN = known; ST = short-term; LT = long-term.

#### Participant P02

The whole brain results for P02 identified significant differences in peak activation for three contrasts of interest in spared left hemisphere regions generally considered to be language-related and in several right hemisphere homologues of those regions. Greater activation for short-term facilitated items was identified in the left pars orbitalis and right pars triangularis of the inferior frontal region, and the bilateral middle temporal poles when compared to known items. Greater activation was also found for long-term items than for known items in several regions of the right hemisphere: the pars orbitalis, the inferior temporal gyrus and the supramarginal gyrus. Finally, a decrease for long-term facilitated items relative to known items was identified in the left middle temporal gyrus.

#### Participant P03

P03 showed only one significant result, with less activation for short-term facilitated than for known items in the right superior temporal pole.

#### Participant P05

The areas of significance for P05 involved a single spared left hemisphere region and several right hemisphere homologues of the language-related network across four contrasts of interest. Greater activation for long-term facilitated items relative to known items was found in the left pars orbitalis. However, for the reverse contrast changes in activation were identified in the right inferior temporal gyrus and right angular gyrus. In addition, the right angular gyrus showed less activity, and the right middle temporal gyrus more activity, for short-term facilitated items relative to known items.

#### Participant P06

The results for P06 identified significant changes in activation for three contrasts across a range of intact left hemisphere language regions and right hemisphere homologous areas. An increase for short-term facilitated items relative to known items was found in the left middle temporal gyrus, the right supramarginal gyrus and the bilateral pars triangularis of the inferior frontal gyrus. Greater activity for long-term facilitated items when compared to known items, however, was identified in the left pars triangularis and left middle temporal gyrus, as well as the right inferior temporal gyrus and right angular gyrus. Additionally, the left pars triangularis also showed greater activation for short-term facilitated items than for long-term facilitated items.

#### Participant P07

Significant differences in activation for P07 were apparent across four contrasts of interest in both left hemisphere language-related regions and right hemisphere homologues. An increase for long-term facilitated items was found in the right superior temporal pole and right middle temporal gyrus relative to known items, and in the bilateral inferior temporal gyri and right superior temporal pole relative to short-term facilitated items. However, a decrease for long-term facilitated items when compared to both known and short-term facilitated items was shown in the bilateral middle temporal gyri and the right pars triangularis.

#### Participant P08

For P08 significant differences were identified in four contrasts of interest across various regions, including left hemisphere language-related areas as well as right hemisphere homologues. An increase for short-term facilitated items relative to known items was shown in the right pars opercularis and right supramarginal gyrus, and a decrease for short-term items relative to known items was observed in the left middle temporal gyrus. For long-term items decreased activity compared to short-term items was found in the right pars opercularis and triangularis, the right middle temporal gyrus, the right angular gyrus and the left pars orbitalis. For the reverse contrast, modulation of activation was identified in the left superior temporal gyrus.

## Discussion

This study investigated the modulation of neural activity associated with the semantic facilitation of picture naming over the short- and long-term in both individuals with aphasia and age-matched controls. Both repetition suppression and repetition enhancement effects were identified. Repetition suppression effects are characterised by a relative decrease in cortical activity following stimulus repetition and are thought to reflect greater processing efficiency [63-65]. Repetition enhancement effects, however, involve an increase in activity and are often associated with additional processing upon repeated stimulus presentation [65,66].

### Control participants

Results for control participants are discussed in detail in Heath et al. [43]. In summary, the behavioral results for control participants showed that a task requiring semantic processing, but which does not include the phonological word form, can result in the short- and long-term behavioral facilitation of picture naming. Further, as Heath et al. [43] report, the whole brain analyses identified a repetition enhancement effect for short-term facilitated items when compared to both unfacilitated and long-term facilitated items. Firstly, an increase in activity for short-term facilitated items was found within the right lingual gyrus (extending into the precuneus region) when compared to unfacilitated items. As the bilateral lingual gyri have been linked to perceptual identification processes and episodic encoding [67], and portions of the precuneus have been associated with mental imagery processes and episodic memory retrieval [68], it may be that episodic encoding or object recognition systems were enhanced during subsequent naming of short-term facilitated items. Increased activity for short-term items when compared to long-term facilitated items was also identified within the left inferior occipital gyrus extending into the left fusiform gyrus, which have been linked to visual association and object recognition [69-71]. Increased activation for short-term facilitated items in this region may, therefore, be due to active visual recognition of the prime picture stimuli presented a few minutes previously. Contrary to our original hypotheses, a lack of modulation of activity in semantic regions suggests that the short-term facilitation effects are primarily driven by object priming and episodic memory mechanisms, rather than more efficient processing at the lexical-semantic level.

### Participants with aphasia

The majority of participants with aphasia (with the exception of P05) showed significantly improved accuracy performance for at least one of the facilitated conditions from baseline to post facilitation (refer to Figure 3 and individual graphs in supplementary material Figure S1). However, for known items that were able to be named prior to facilitation, performance decreased significantly during the scanning session. This decrease could reflect regression to the mean. As the known condition was purposefully selected from items able to be named over two previous pre-tests, it is possible that performance simply regressed towards the mean during subsequent exposure, resulting in decreased accuracy. This phenomenon could similarly account for the increased accuracy for facilitated conditions within the scanner, as these were chosen from consistently difficult to name items. This possibility is reduced, however, due to the fact that the stress associated with performing a task within the scanner is known to have a negative impact upon behavioral measures [72,73], as shown by the generally improved performance at the follow-up session conducted outside the scanner. Despite this, caution is needed when considering the significant improvements in naming accuracy for facilitated conditions identified in the current study.

With this caution in mind, it is noteworthy that the two participants with the lowest percentage accuracy during performance of the semantic facilitation task (P03 and P08) were the only two individuals to show significant improvements in naming following both short-term and long-term facilitation (refer to Figure S1). While poor performance of the facilitation task for P03 and P08 was likely influenced by auditory comprehension difficulties (refer to Table 2), it is interesting that the other four participants all displayed more semantic than phonological naming errors during pre-test sessions. This pattern across behavioral results highlights the fact that semantic naming errors may not always be indicative of semantic processing deficits and further, could suggest that individuals with semantic processing deficits (shown in the current study as poor performance during the facilitation task) would benefit the most from semantically-focused treatments.

With regard to the whole brain imaging analyses (refer to supplementary material Table S1), significant results appear to be more extensive for participants with aphasia than for healthy controls. This may be due to less conservative thresholding (*p* < 0.01 as opposed to *p* < 0.001) for these individuals, or could reflect increased difficulty or effort required to successfully complete the task on the part of individuals with aphasia. Regardless, it is clear that these participants did not show similar results to the control group while performing exactly the same task. Only P05 and P08 had significant changes in activation within the same regions as controls (right lingual gyrus and right precuneus). However, these changes were not identified for the same contrasts of interest as the control group. Overall, different mechanisms appear to underlie the facilitation of naming with a semantic task for the individuals with aphasia participating in this study, when compared to the control group. This finding is in contrast to other studies proposing that the patterns of activation for accurate naming in aphasia are similar to that found in healthy controls [74,75]. Importantly, these studies did not specifically examine activity associated with the facilitation of naming in both controls and in aphasia. It is also evident from the present investigation that the participants with aphasia were not engaging a network associated exclusively with semantics. P03 and P05 were the only participants to show modulation of activity for contrasts of interest restricted to regions linked to semantic processing, and P05 did not show significant behavioral change in naming. Further, in line with previous research [14,76] the whole brain results highlight the involvement of both non-linguistic and subcortical areas in the successful facilitation of naming.

The relationship between performance and patterns of neural activation in this group of individuals with aphasia was not straightforward. Each participant displayed a different pattern of activity, recruiting a combination of spared left hemisphere language-related regions, and the right hemisphere homologues of both lesioned and intact regions of the normal language network. With the exception of P03 and P05, differences in activation for specific contrasts were identified in some phonological regions for participants. The vast majority of results, however, were found within either left hemisphere regions across the frontal and temporal lobes associated with semantic processing, or their right hemisphere homologues and the right inferior parietal region. In contrast to control participants, the facilitation of naming in this group of individuals with aphasia appeared to be predominantly driven by fronto-temporal mechanisms associated with semantics over both the short- and the long-term.

The following discussion of results from the whole brain analyses focuses on the most significant area of peak activation (based on largest cluster and/or highest Z-score) located within language-related regions, or their right hemisphere homologues (refer to Figure 4 and highlighted results in Table 4). It has been assumed that the most significant results for contrasts of interest are likely to represent the primary mechanism underlying facilitation for each participant. Our proposals regarding primary mechanisms of facilitation, therefore, need to be interpreted with some caution and with reference to the full reported results (see supplementary material Table S1). Additionally, some participants showed minimal differences in activity for contrasts within language-related regions and are underrepresented.

#### Changes in activation within regions associated with phonology

When considering the summarised results, only one participant showed the most significant differences of activation for contrasts of interest in regions linked to phonological processing. For P08 a decrease in activation for short-term facilitated items relative to long-term items was identified in the left superior temporal gyrus, and an increase for short-term items when compared to known items in the right pars opercularis. The majority of significant results, however, fall within areas linked to semantic processing or their right hemisphere homologues, with minimal involvement of phonological regions. This pattern is consistent with the hypothesis that the semantic facilitation task can successfully target the lexical-semantic level of processing to improve subsequent naming. Additionally, this isolated example of differences in activation within phonological areas could indicate that P08 was applying some form of phonological processing strategy (e.g. subvocal rehearsal and/or monitoring) to the pictures she was naming, which differed as a function of facilitation [77]. The pre-test error breakdown (see Table 2) provides some support for this suggestion. P08 made a substantial number of phonological errors during the pre-test naming batteries and may have experienced increased demands upon, or increased effort required to engage, phonological processes for accurate naming than the other participants. Further, as P08 made a large number of overall errors during the pre-tests and facilitation sessions and has one of the lowest aphasia quotient scores [45], modulation of activity in areas linked to phonological processing for this participant may simply relate to the severity of impairment to the underlying processes involved in naming.

#### Changes in activation within regions associated with semantics

Five participants (P02, P03, P05, P06 and P07) utilized spared left hemisphere regions linked to semantic processing as the primary mechanism underlying facilitation for several contrasts of interest. These areas included the left middle temporal gyrus for P02 and P07, and the pars orbitalis and pars triangularis of the left inferior frontal gyrus for P05 and P06 respectively (refer to Table 4 for specific contrasts). The only participant showing significant changes in activation within a left hemisphere area of lesion involvement was P08, with relatively less activation for short-term items than for known items in the left middle temporal gyrus. Previous studies have shown that perilesional activity is associated with good recovery of language in the chronic stage [31,35,70,75]. The current study, however, found that modulation of activity close to lesioned regions was not critical in this group of subjects. Other studies have suggested that individuals with smaller lesions rely on spared perilesional language areas [14]. Providing limited support for this suggestion is the finding that P08, who had the smallest lesion volume of the six participants, was the only individual to show significant changes in activation within a lesioned area. However, this was not the case for P05 with a comparable lesion volume.

Right-hemisphere engagement was also identified for all participants within regions homologous to those associated with semantics. Previous research has shown that relateralization of activity to the right hemisphere generally occurs within regions homologous to lesioned cortical structures [78-81]. Conversely, in this group only two participants (P02 and P05) showed activation within right hemisphere homologues of lesioned regions as the primary mechanism underlying facilitation. Greater activation for short-term facilitated items was identified when compared to known items in the right pars triangularis for P02 and for the same contrast in the right middle temporal gyrus for P05. All participants, however, showed significant differences in right hemisphere activation across a range of contrasts involving language-related homologues that were spared in the left hemisphere. These right hemisphere regions included the inferior temporal gyrus for P02 and P07, the angular gyrus for P05 and P06, the middle temporal gyrus for P07, and the pars triangularis of the inferior frontal gyrus for P08 (refer to Table 4 for specific contrasts). In the case of P03, who had the most difficulty performing the semantic task during facilitation (see Table 2), less activation for short-term facilitated items than for known items was identified in the right hemisphere superior temporal pole. Evidence suggests that the temporal pole is part of a bilateral semantic system supporting representations of object concepts and mapping concepts to words during production [82,83]. This repetition suppression effect for short-term facilitated items in the right hemisphere represented the only significant result for P03 in a language-related homologue.

## Conclusions

This study utilized a unique methodology to examine the effects of a specific semantic facilitation technique on subsequent successful picture naming performance in people with aphasia and age-matched controls. Behavioral results showed that a semantic task that does not include the phonological word form may successfully facilitate subsequent picture naming for healthy controls and some individuals with aphasia. However, the neurocognitive mechanisms underlying these effects appeared to differ between controls and participants with aphasia. Although a repetition enhancement effect was found for control subjects in the short-term, contrary to our original hypothesis, modulation of activity was not identified in regions traditionally associated with semantic processing. The results instead suggested that short-lived facilitation effects were primarily driven by object priming and episodic memory mechanisms, rather than more efficient processing at the lexical-semantic level.

Results for individuals with aphasia did not mirror those observed in the control group and were less definitive. Although most participants showed some improvement in accuracy for facilitated items, different patterns of activation were evident for each individual. It was hypothesized that facilitation effects for the participants with aphasia would predominantly engage a semantic network. In this regard, the results did identify a more extensive pattern of activation in semantic regions in comparison to the control group. This observation provides evidence that a prior semantic task, in the absence of the word form, may positively influence subsequent naming at the level of lexical-semantics over both the short- and the long-term for participants with aphasia. The results of the present study also indicate that right hemisphere involvement may be supportive of naming facilitation rather than maladaptive. Further, in contrast to previous assumptions, modulation of activity close to lesioned regions in the left hemisphere, and in the right hemisphere homologues of lesioned regions, did not appear to be critical for this group of participants. Finally, the results also highlight the involvement of a distributed perisylvian network in both hemispheres, as well as subcortical and cerebellar regions, in the facilitation of picture naming.

Although limited claims can be made regarding specific mechanisms of recovery on the basis of the current results, our findings do highlight the utility of this unique methodology. Larger scale studies utilizing a similar paradigm and including participants with aphasia who can be grouped according to clinical profile (e.g. individuals with apparent semantic deficits), or site and size of lesion, may provide more definitive results regarding specific underlying mechanisms. Further investigation of the neurocognitive substrates of naming facilitation using fMRI may provide evidence regarding selection of the best approach to treatment of anomia for specific individuals, and could ultimately aid in the development of more theoretically driven and neurobiologically informed treatment methods.

## Competing interests

The authors declare that they have no competing interests.

## Authors' contributions

SH contributed to study design, performed behavioral and neuroimaging testing, conducted data analyses and interpretation, and wrote the manuscript. KM contributed to the design of the study, oversaw neuroimaging data collection, contributed to analyses and interpretation, and revised the manuscript. LN contributed to study design, assisted with interpretation of results and revised the manuscript critically for intellectual content. AA helped to design the study and revise the manuscript for intellectual content. AM contributed to study design, assisted with data collection and analysis, as well as helped to revise the manuscript. SvH, KJ and EM were involved with data collection and analysis. DC was responsible for the conceptualization and design of the study, contributed to statistical analyses, assisted with interpretation of results and revised the manuscript critically for important intellectual content. All authors read and approved the final manuscript.

## Supplementary Material

Additional file 1 **Figure S1.**Facilitation effects in accuracy data for participants with aphasia. Individual graphs showing facilitation effects in percentage accuracy data for all conditions. LT = long-term facilitated; ST = short-term facilitated; KN = known (and unfacilitated). * indicates a significant difference (p < 0.05) between pre-facilitation (Pre-Test 1 and Pre-Test 2) and post-facilitation (In-Scan and Follow-Up) percentage accuracy scores for each condition. Click here for file

Additional file 2 **Table S1.** Figure S1.Facilitation effects in accuracy data for participants with aphasia. Individual graphs showing facilitation effects in percentage accuracy data for all conditions. LT = long-term facilitated; ST = short-term facilitated; KN = known (and unfacilitated). * indicates a significant difference (p < 0.05) between pre-facilitation (Pre-Test 1 and Pre-Test 2) and post-facilitation (In-Scan and Follow-Up) percentage accuracy scores for each condition. Click here for file
