# Supplementary figures and images for: Neural mechanisms underlying the facilitation of naming in aphasia using a semantic task: an fMRI study
Source: BMC Neurosci. 2012 Aug 10;13:98. doi: 10.1186/1471-2202-13-98 (PMC3477078; doi:10.1186/1471-2202-13-98)

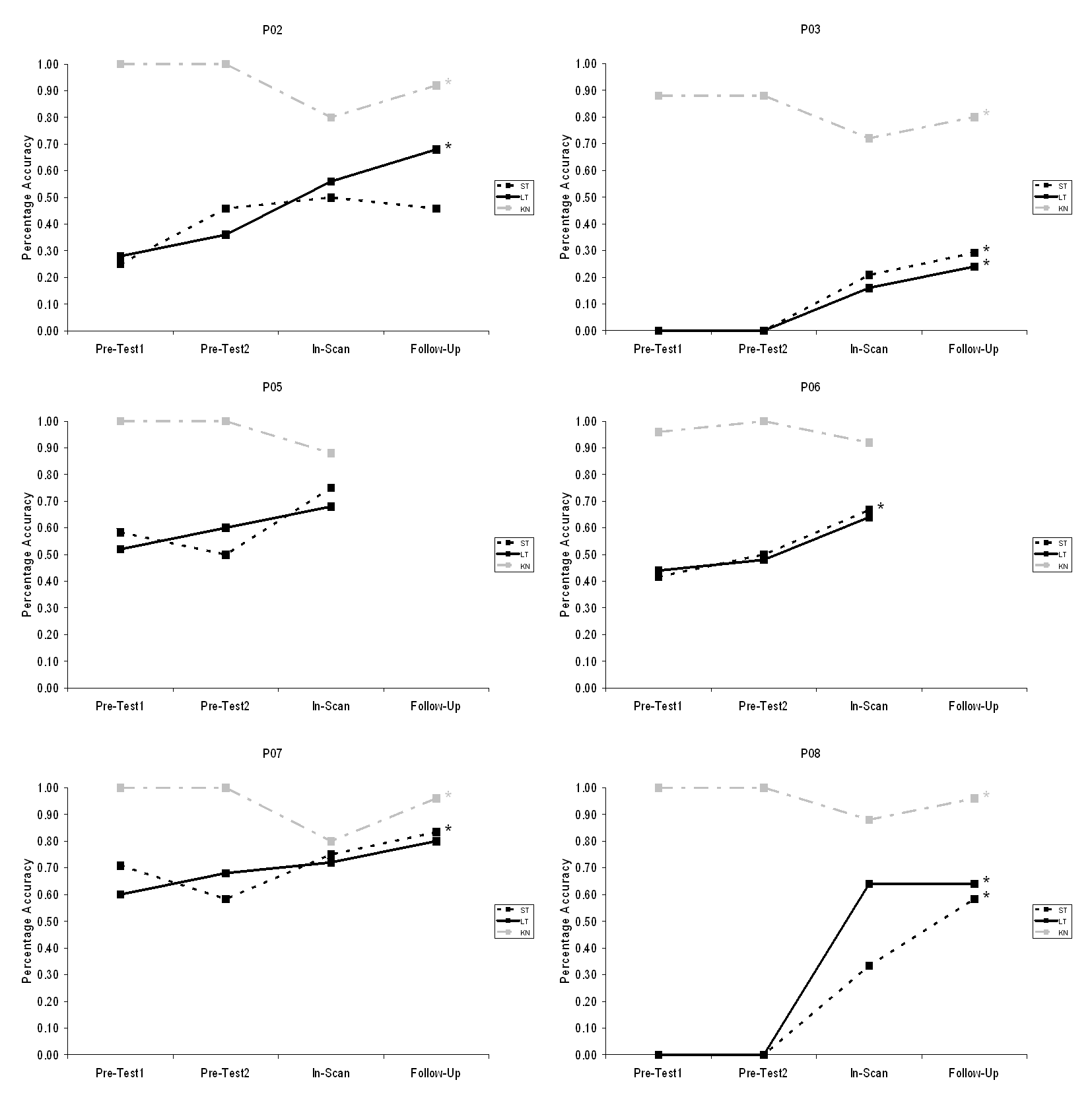

Supplement: Additional file 1 — Figure S1.Facilitation effects in accuracy data for participants with aphasia. Individual graphs showing facilitation effects in percentage accuracy data for all conditions. LT = long-term facilitated; ST = short-term facilitated; KN = known (and unfacilitated). * indicates a significant difference (p < 0.05) between pre-facilitation (Pre-Test 1 and Pre-Test 2) and post-facilitation (In-Scan and Follow-Up) percentage accuracy scores for each condition. [file 1471-2202-13-98-S1.tiff]
