# Supplementary material for: Neural mechanisms underlying the facilitation of naming in aphasia using a semantic task: an fMRI study
Source: BMC Neurosci. 2012 Aug 10;13:98. doi: 10.1186/1471-2202-13-98 (PMC3477078; doi:10.1186/1471-2202-13-98)
Supplement: Additional file 2 — Table S1. Figure S1.Facilitation effects in accuracy data for participants with aphasia. Individual graphs showing facilitation effects in percentage accuracy data for all conditions. LT = long-term facilitated; ST = short-term facilitated; KN = known (and unfacilitated). * indicates a significant difference (p < 0.05) between pre-facilitation (Pre-Test 1 and Pre-Test 2) and post-facilitation (In-Scan and Follow-Up) percentage accuracy scores for each condition. [file 1471-2202-13-98-S2.doc]

| **Contrast and Anatomical Label** | **Hem** | **Vol** | **x** | **y** | **z** | **Z** |
| --- | --- | --- | --- | --- | --- | --- |
| **P02** |  |  |  |  |  |  |
| Short-Term > Known: |  |  |  |  |  |  |
| - temporal pole (middle) | L | 21 | -45 | 12 | -39 | 4.29 |
| - inferior frontal gyrus (triangularis) extending to inferior frontal gyrus (opercularis) and superior frontal gyrus (orbital part) | R | 268 | 42 | 33 | 12 | 3.73 |
| - postcentral gyrus | R | 67 | 42 | -36 | 60 | 3.50 |
| - temporal pole (middle) extending to fusiform gyrus | R | 34 | 36 | 6 | -39 | 3.30 |
| - middle cingulum | R | 47 | 9 | 18 | 30 | 3.20 |
| - inferior parietal gyrus | L | 23 | -60 | -33 | 51 | 3.07 |
| - inferior frontal gyrus (orbitalis) | L | 23 | -45 | 33 | -9 | 2.90 |
| Known > Short-Term: |  |  |  |  |  |  |
| - superior frontal gyrus extending to superior frontal gyrus (medial part) | R | 43 | 12 | 72 | 21 | 3.10 |
| - lingual gyrus | L | 31 | -18 | -81 | -3 | 2.88 |
| Long-Term > Known: |  |  |  |  |  |  |
| - superior frontal gyrus (orbital part) extending to inferior frontal gyrus (opercularis) and precentral gyrus | R | 388 | 21 | 60 | -9 | 4.12 |
| - inferior temporal gyrus extending to temporal pole (middle) and fusiform gyrus | R | 34 | 39 | -3 | -45 | 3.92 |
| - inferior parietal gyrus | L | 37 | -48 | -45 | 48 | 3.53 |
| - superior parietal gyrus extending to inferior parietal gyrus | L | 176 | -27 | -60 | 57 | 3.47 |
| - inferior frontal gyrus (orbitalis) | R | 22 | 42 | 42 | -9 | 3.21 |
| - hippocampus | R | 32 | 39 | -21 | -15 | 2.99 |
| - supramarginal gyrus | R | 28 | 48 | -33 | 39 | 2.69 |
| - postcentral gyrus | R | 31 | 30 | -33 | 42 | 2.63 |
| - middle cingulum | R | 29 | 15 | -36 | 33 | 2.54 |
| Known > Long-Term: |  |  |  |  |  |  |
| - supplementary motor area extending to left precentral gyrus | R | 809 | 3 | 9 | 54 | 3.77 |
| - middle temporal gyrus | L | 25 | -66 | -30 | 0 | 3.28 |
| - hippocampus | L | 22 | -21 | -24 | -12 | 3.28 |
| - postcentral gyrus | L | 20 | -57 | -9 | 48 | 3.12 |
| - middle frontal gyrus | R | 21 | 48 | 3 | 57 | 2.85 |
| Short-Term > Long-Term: |  |  |  |  |  |  |
| - inferior parietal gyrus | L | 23 | -60 | -27 | 48 | 3.39 |
| - insula | L | 34 | -36 | 3 | -9 | 3.22 |
| - precentral gyrus extending to postcentral gyrus | L | 43 | -57 | -3 | 33 | 3.07 |
| - precentral gyrus | L | 34 | -42 | 3 | 42 | 2.69 |
| Long-Term > Short-Term: |  |  |  |  |  |  |
| - middle occipital gyrus | L | 20 | -27 | -78 | 3 | 3.06 |
| **P03** |  |  |  |  |  |  |
| Short-Term > Known: |  |  |  |  |  |  |
| - middle frontal gyrus | R | 28 | 39 | 27 | 21 | 2.98 |
| Known > Short-Term: |  |  |  |  |  |  |
| - temporal pole (superior) extending to temporal pole (middle) | R | 53 | 36 | 6 | -24 | 3.20 |
| - superior temporal gyrus extending to insula | R | 35 | 57 | 0 | -12 | 3.03 |
| Long-Term > Known: |  |  |  |  |  |  |
| - no significant results |  |  |  |  |  |  |
| Known > Long-Term: |  |  |  |  |  |  |
| - no significant results |  |  |  |  |  |  |
| Short-Term > Long-Term: |  |  |  |  |  |  |
| - cerebellum X | L | 22 | 0 | -18 | -45 | 2.91 |
| Long-Term > Short-Term: |  |  |  |  |  |  |
| - cerebellum crus 1 extending to cerebellum VI and VIII | L | 82 | -27 | -63 | -39 | 3.03 |
| - supplementary motor area | L | 21 | -9 | -18 | 51 | 2.89 |
| **P05** |  |  |  |  |  |  |
| Short-Term > Known: |  |  |  |  |  |  |
| - superior frontal gyrus extending to middle frontal gyrus | R | 466 | 21 | 51 | 27 | 3.90 |
| - superior frontal gyrus (medial part) extending to middle frontal gyrus | L | 158 | -6 | 39 | 30 | 3.21 |
| - supplementary motor area | L | 26 | -3 | -3 | 54 | 3.20 |
| - middle temporal gyrus | R | 20 | 60 | -6 | -24 | 2.89 |
| Known > Short-Term: |  |  |  |  |  |  |
| - angular gyrus extending to precuneus | R | 299 | 36 | -72 | 42 | 3.48 |
| - calcarine fissure | R | 25 | 27 | -75 | 12 | 2.92 |
| - inferior parietal gyrus extending to superior parietal gyrus | R | 26 | 51 | -48 | 54 | 2.68 |
| Long-Term > Known: |  |  |  |  |  |  |
| - middle frontal gyrus extending to superior frontal gyrus (medial part) | R | 152 | 24 | 51 | 27 | 3.36 |
| - inferior frontal gyrus (orbitalis) | L | 42 | -42 | 27 | -9 | 3.12 |
| - superior frontal gyrus extending to superior frontal gyrus (medial part) | L | 45 | -15 | 51 | 30 | 3.08 |
| Known > Long-Term: |  |  |  |  |  |  |
| - precuneus | L | 124 | -3 | -66 | 57 | 3.89 |
| - angular gyrus | R | 105 | 36 | -72 | 42 | 3.38 |
| - calcarine fissure extending to superior occipital gyrus | R | 46 | 24 | -75 | 9 | 3.27 |
| - cuneus extending to left precuneus | R | 87 | 12 | -69 | 24 | 3.02 |
| - angular gyrus | R | 26 | 60 | -57 | 30 | 2.97 |
| - inferior temporal gyrus | R | 20 | 39 | -54 | -3 | 2.97 |
| - lingual gyrus extending to cerebellum crus 1 | R | 28 | 39 | -78 | -18 | 2.84 |
| Short-Term > Long-Term: |  |  |  |  |  |  |
| - no significant results |  |  |  |  |  |  |
| Long-Term > Short-Term: |  |  |  |  |  |  |
| - no significant results |  |  |  |  |  |  |
| **P06** |  |  |  |  |  |  |
| Short-Term > Known: |  |  |  |  |  |  |
| - inferior frontal gyrus (triangularis) | L | 130 | -45 | 27 | 21 | 3.69 |
| - inferior frontal gyrus (triangularis) | R | 25 | 45 | 36 | 12 | 3.58 |
| - calcarine fissure | R | 52 | 21 | -96 | 0 | 3.52 |
| - fusiform gyrus extending to middle temporal gyrus and cerebellum VI | R | 517 | 42 | -66 | -18 | 3.49 |
| - middle temporal gyrus extending to inferior temporal gyrus | L | 144 | -54 | -69 | 18 | 3.42 |
| - precentral gyrus | L | 28 | -42 | -6 | 57 | 3.21 |
| - postcentral gyrus extending to paracentral lobule | R | 30 | 15 | -39 | 75 | 3.20 |
| - middle occipital gyrus extending to superior occipital gyrus | R | 34 | 30 | -66 | 36 | 3.06 |
| - middle occipital gyrus | L | 34 | -36 | -90 | 6 | 2.95 |
| - precentral gyrus extending to inferior frontal gyrus (opercularis) | R | 27 | 45 | 6 | 27 | 2.93 |
| - supramarginal gyrus | R | 24 | 60 | -39 | 39 | 2.80 |
| Known > Short-Term: |  |  |  |  |  |  |
| - cuneus extending to precuneus | L | 53 | -18 | -57 | 30 | 3.32 |
| - middle frontal gyrus | L | 44 | -21 | 18 | 33 | 3.11 |
| - precuneus | R | 25 | 15 | -54 | 30 | 2.95 |
| - putamen extending to insula | L | 25 | -30 | -3 | 6 | 2.92 |
| - insula extending to inferior frontal gyrus (orbitalis) | R | 28 | 30 | 21 | -12 | 2.91 |
| - middle cingulum | L | 27 | 0 | -33 | 42 | 2.84 |
| Long-Term > Known: |  |  |  |  |  |  |
| - inferior temporal gyrus extending to middle temporal gyrus | R | 47 | 42 | -54 | -3 | 3.86 |
| - angular gyrus extending to superior occipital gyrus | R | 63 | 30 | -51 | 42 | 3.38 |
| - inferior frontal gyrus (triangularis) | L | 33 | -51 | 36 | 6 | 3.13 |
| - middle temporal gyrus extending to angular gyrus | L | 34 | -54 | -72 | 18 | 3.07 |
| Known > Long-Term: |  |  |  |  |  |  |
| - insula | R | 23 | 33 | 21 | -9 | 2.97 |
| Short-Term > Long-Term: |  |  |  |  |  |  |
| - rolandic operculum | R | 36 | 60 | -12 | 15 | 3.58 |
| - inferior frontal gyrus (triangularis) extending to inferior frontal gyrus (opercularis) and precentral gyrus | L | 56 | -42 | 27 | 21 | 3.27 |
| - cerebellum crus 1 extending to inferior occipital gyrus and fusiform gyrus | R | 54 | 36 | -75 | -27 | 3.22 |
| - calcarine | L | 45 | -12 | -87 | 3 | 2.97 |
| - cerebellum VI extending to lingual gyrus | L | 126 | -15 | -72 | -21 | 2.87 |
| Long-Term > Short-Term: |  |  |  |  |  |  |
| - superior occipital gyrus extending to cuneus and right precuneus | L | 662 | -21 | -63 | 30 | 4.31 |
| - cerebellum IX extending to IV and V | R | 34 | 18 | -45 | -36 | 3.25 |
| - middle frontal gyrus extending to caudate nucleus | L | 25 | -21 | 18 | 30 | 3.17 |
| - postcentral gyrus extending to superior parietal gyrus | R | 38 | 18 | -30 | 60 | 2.96 |
| - superior frontal gyrus (medial part) extending to middle frontal gyrus (orbital part) | L | 21 | -12 | 66 | 12 | 2.87 |
| **P07** |  |  |  |  |  |  |
| Short-Term > Known: |  |  |  |  |  |  |
| - no significant results |  |  |  |  |  |  |
| Known > Short-Term: |  |  |  |  |  |  |
| - middle frontal gyrus | L | 54 | -36 | 51 | 0 | 3.23 |
| Long-Term > Known: |  |  |  |  |  |  |
| - temporal pole (superior) | R | 25 | 36 | 21 | -27 | 3.31 |
| - middle temporal gyrus extending to temporal pole (middle) | R | 35 | 54 | 6 | -30 | 3.19 |
| - caudate nucleus | L | 21 | -21 | -27 | 24 | 3.08 |
| - precentral gyrus | R | 44 | 27 | -21 | 66 | 2.88 |
| - paracentral lobule | L | 27 | -12 | -18 | 72 | 2.73 |
| Known > Long-Term: |  |  |  |  |  |  |
| - middle temporal gyrus | L | 71 | -57 | -48 | -6 | 3.96 |
| - middle frontal gyrus (orbital part) extending to inferior frontal gyrus (orbitalis) | L | 23 | -33 | 45 | -12 | 3.33 |
| - cerebellum VIII | R | 28 | 39 | -42 | -48 | 3.32 |
| - inferior frontal gyrus (triangularis) extending to insula and caudate nucleus | R | 26 | 39 | 33 | 12 | 3.26 |
| - middle frontal gyrus | R | 32 | 39 | 48 | 30 | 3.14 |
| - anterior cingulum | L | 38 | -18 | 36 | 15 | 3.10 |
| - middle temporal gyrus | R | 52 | 57 | -51 | -9 | 3.09 |
| - rolandic operculum | R | 28 | 45 | 9 | 15 | 2.99 |
| - anterior cingulum | R | 21 | 18 | 42 | 21 | 2.91 |
| - cuneus extending to right cuneus | L | 20 | -6 | -93 | 33 | 2.76 |
| Short-Term > Long-Term: |  |  |  |  |  |  |
| - middle temporal gyrus | L | 116 | -48 | -45 | -3 | 4.09 |
| - inferior frontal gyrus (triangularis) extending to inferior frontal gyrus (opercularis) | R | 81 | 51 | 18 | 24 | 3.72 |
| - middle frontal gyrus extending to insula and inferior frontal gyrus (opercularis) | R | 54 | 39 | 36 | 12 | 3.43 |
| - middle temporal gyrus | R | 25 | 57 | -39 | 0 | 3.25 |
| - cuneus | L | 33 | 3 | -87 | 30 | 2.95 |
| Long-Term > Short-Term: |  |  |  |  |  |  |
| - inferior temporal gyrus extending to amygdala and temporal pole (superior) | R | 116 | 45 | -6 | -33 | 3.71 |
| - temporal pole (superior) | R | 33 | 36 | 21 | -27 | 3.56 |
| - heschl gyrus | R | 32 | 57 | -6 | 6 | 3.10 |
| - inferior temporal gyrus | L | 28 | -36 | -3 | -36 | 3.00 |
| - calcarine fissure extending to fusiform gyrus | R | 24 | 24 | -87 | 0 | 2.90 |
| **P08** |  |  |  |  |  |  |
| Short-Term > Known: |  |  |  |  |  |  |
| - supramarginal gyrus extending to superior temporal gyrus | R | 67 | 63 | -36 | 27 | 3.54 |
| - inferior frontal gyrus (opercularis) extending to rolandic operculum and postcentral gyrus | R | 141 | 57 | 18 | -3 | 3.36 |
| - anterior cingulum extending to superior frontal gyrus (orbital part) and middle frontal gyrus (orbital part) | L | 99 | -3 | 33 | -9 | 3.02 |
| Known > Short-Term: |  |  |  |  |  |  |
| - calcarine fissure extending to superior occipital gyrus | R | 51 | 33 | -66 | 12 | 3.20 |
| - middle temporal gyrus extending to calcarine fissure and superior temporal gyrus | L | 117 | -36 | -48 | 18 | 3.14 |
| - caudate nucleus | L | 20 | -18 | 30 | 12 | 3.05 |
| Long-Term > Known: |  |  |  |  |  |  |
| - parahippocampal gyrus | R | 29 | 18 | -21 | -36 | 3.04 |
| Known > Long-Term: |  |  |  |  |  |  |
| - putamen | L | 26 | -21 | -3 | 12 | 2.62 |
| Short-Term > Long-Term: |  |  |  |  |  |  |
| - inferior frontal gyrus (orbitalis) | L | 60 | -48 | 30 | -12 | 3.79 |
| - anterior cingulum extending to right anterior cingulum and left middle frontal gyrus (orbital part) | L | 199 | -3 | 30 | -9 | 3.51 |
| - inferior frontal gyrus (triangularis) extending to inferior frontal gyrus (opercularis) | R | 69 | 57 | 21 | -3 | 3.46 |
| - middle frontal gyrus extending to inferior frontal gyrus (opercularis) | L | 39 | -39 | 9 | 33 | 3.42 |
| - inferior frontal gyrus (opercularis) | R | 89 | 45 | 15 | 27 | 3.30 |
| - lingual gyrus extending to fusiform gyrus | R | 185 | 24 | -84 | -6 | 3.26 |
| - angular gyrus | R | 20 | 33 | -60 | 51 | 3.24 |
| - putamen | L | 78 | -21 | 9 | 3 | 3.15 |
| - precentral gyrus | L | 33 | -39 | 3 | 48 | 3.12 |
| - middle temporal gyrus | R | 29 | 57 | -30 | -6 | 2.97 |
| - lingual gyrus | L | 20 | -24 | -63 | -12 | 2.86 |
| - middle temporal gyrus extending to superior temporal gyrus | R | 24 | 69 | -42 | 9 | 2.82 |
| Long-Term > Short-Term: |  |  |  |  |  |  |
| - middle frontal gyrus extending to superior frontal gyrus | L | 43 | -27 | 57 | 18 | 3.67 |
| - superior temporal gyrus extending to angular gyrus and middle temporal gyrus | L | 162 | -39 | -36 | 12 | 3.53 |
| - superior frontal gyrus (medial part) | R | 36 | 12 | 63 | 6 | 3.49 |
| - middle frontal gyrus extending to superior frontal gyrus | R | 67 | 39 | 48 | 30 | 3.34 |
| - calcarine fissure extending to middle temporal gyrus | R | 78 | 33 | -66 | 12 | 3.20 |
| - cerebellum X | L | 29 | -12 | -27 | -42 | 3.12 |
| - middle frontal gyrus extending to superior frontal gyrus | L | 36 | -33 | 42 | 39 | 3.05 |
